# Editorial: New discoveries in the benefits and outcomes of cochlear implantation

**DOI:** 10.3389/fnins.2022.1062582

**Published:** 2022-11-15

**Authors:** Fei Chen, Jing Chen, Xin Luo

**Affiliations:** ^1^Department of Electrical and Electronic Engineering, Southern University of Science and Technology, Shenzhen, China; ^2^Key Laboratory of Machine Perception, Ministry of Education, School of Intelligence Science and Technology, College of Future Technology, Peking University, Beijing, China; ^3^Speech and Hearing Science, College of Health Solutions, Arizona State University, Tempe, AZ, United States

**Keywords:** cochlear implantation, speech perception, cochlear implant benefits, psychophysical and neurophysiological methods, music and emotion perception

Cochlear implants (CIs), hearing prostheses that bypass sensory hair cells in the cochlea to directly stimulate the auditory nerve, have been shown to restore hearing for individuals with severe to profound hearing loss. Recent research has demonstrated enormous CI benefits for speech recognition, sound localization, as well as language development in children. The effectiveness of CIs is, however, affected by many factors, including the duration of deafness/hearing loss, implantation age, and duration of CI use. Whereas recent studies have largely focused on the effects of CIs on speech perception and production, further investigation is needed to study the effects of CIs on emotion processing, music perception, prosody perception, etc. The aim of this Research Topic Collection is to bring together studies addressing recent discoveries in the benefits and outcomes of cochlear implantation. A total of 17 papers are included in this Research Topic.

Five papers utilized neuroimaging techniques, including electroencephalogram (EEG) and functional near-infrared spectroscopy (fNIRS), to investigate CI users' cortical response characteristics on a range of auditory perception tasks. (1) As P300 is closely related to cognitive processes (e.g., auditory discrimination), Tao et al. investigated the connection between auditory segregation of competing speech in Mandarin-speaking bimodal CI users and the P300 component of event-related potentials elicited by 1 vs. 2 kHz contrast. Their results showed that the P300 amplitude was significantly correlated with the speech reception threshold in the same target-masker voice gender (male) condition in the CI group, which suggests the potential of P300 amplitude as a clinically useful neural indicator of central auditory processing capabilities that are susceptible to informational masking in bimodal CI users. (2) Xie et al. explored the relationship between the ability to detect frequency changes or temporal gaps and speech perception using psychophysical and neurophysiological methods among post-lingually deafened CI users. Their multiple regression analysis showed the predictive ability of gap detection threshold (GDT) and the amplitude of acoustic change complex (ACC) response on speech perception performance. This indicates that GDT (as a psychophysical measure) may work as an easy, quick and non-linguistic tool, and ACC amplitude induced by the temporal gap (as a neurophysiological indicator) may have the potential to predict speech outcomes. (3) Cartocci et al. studied the emotion processing of children with unilateral CI *via* EEG and behavioral measures. Compared to normal-hearing (NH) children, less accurate vocal emotional state recognition was observed in children with unilateral CI, which was correlated with increased gamma activity lateralization index (relatively higher right-hemisphere activity) in response to emotional speech stimuli. The implantation side for children with unilateral CI did not affect the contralateral gamma activity, but the age at implantation influenced emotion recognition. These indicate that a deficit in engaging the left hemisphere for emotional tasks exists in unilateral CI users and early implantation may be beneficial to children's emotion recognition. (4) Raghavendra et al. investigated the sound quality of speech produced by CI users from the perspective of NH listeners' perception. They decoded and re-constructed the speech envelope from single-trial EEG recorded on the scalp of the NH listeners using a regenerative model and computed the correlation between the actual and reconstructed speech envelope waveforms. They found that the perceived sound quality rating was associated with the cortical tracking of speech envelop, and speech produced by NH speakers was more closely tracked relative to that produced by CI speakers. (5) Lu et al. focused on the cortical coding of prosody measured by fNIRS in pre-lingually deafened children with CIs. They recorded cortical responses to natural sentences with strong or weak prosodic features, and evaluated participants' speech communication ability in three tasks of picture description, video content statement and free conversation. Weaker cortical activation and characteristic deficits in perceiving strong prosodies were observed in children with CIs compared to NH children. Sensitivity to strong prosodic information was significantly correlated with the speech communication ability of all children who participated in this study. This suggests the importance of speech prosody in children's speech development.

Understanding the predictive factors of CI users' auditory performance is crucial to prognosis and clinical decision-making. Factors that have reached general consensus such as age at implantation and duration of device use cannot explain all the variance in CI performance; exploring new independent factors that have predicative power is thus needed. (1) Lu et al. retrospectively examined clinical results of children with cochlear nerve deficiency (CND) and CIs in order to identify main predictive factors and develop predictive models using machine learning. A parameter-optimized support vector machine based on the vestibulocochlear nerve (VCN) area and the number of nerve bundles (measured with high-resolution computed tomography) was constructed to predict speech perception performance. These two factors were suggested to have the ability to predict CI outcomes in children with CND (i.e., prediction accuracies of 71 and 93% in hearing rehabilitation and speech rehabilitation, respectively), providing guidance on surgical side selection and prognosis. (2) Chao et al. focused on the predictive ability of pre-implantation imaging results for electrically evoked compound action potentials (ECAPs) of auditory nerve fibers in response to electrical stimuli in children with CND. They found that the width of the bony cochlear nerve canal or the VCN diameter did not correlate with ECAP responses, while the ratio of the VCN to facial nerve (FN) diameter was significantly correlated with the slope of the ECAP input/output function and the ECAP maximum amplitude. This suggests that the VCN to FN diameter ratio may be an effective predictor of the cochlear nerve function in children with CND and CIs. (3) Zheng and Liu reviewed CI outcomes in patients with auditory neuropathy caused by Otoferlin (OTOF) gene mutations. They concluded that patients with OTOF mutations had excellent performance in both sound perception and speech recognition, and suggested the importance of genetic analysis in localizing lesions and informing clinical decision-making. Early implantation for patients with biallelic OTOF mutations was encouraged. (4) Zhang et al. investigated the predictive power of imaging results but for the long-term auditory and speech perception development of pediatric CI users with common cavity deformity (CCD). Auditory and speech behaviors [using four parent reports questionnaires: categories of Auditory Performance (CAP), Speech Intelligibility Rating (SIR), Meaningful Auditory Integration Scale/Infant-Toddler Meaningful Auditory Integration Scale (MAIS/ITMAIS), and Meaningful Use of Speech Scale (MUSS)] improved over time after implantation in children with CCD, but were poorer than those of CI users with normal inner ear structures. The volume and lumen surface range of CCD reflected inner ear development and influenced CI outcomes.

The remaining eight papers cover a broad range of new topics on the outcomes of cochlear implantation. (1) Mao et al. themed around Mandarin tone production of pre-lingually deafened children with CIs. They recorded monosyllables produced by each participant and calculated the differentiability and hit rate of different tones using acoustic analyses. In general, children with CIs exhibited significantly poorer tone productions in both measures compared with NH children. A weak correlation between age at implantation or duration of CI use and acoustic measures (i.e., differentiability and hit rate, computed based on the F0 onset and offset values or the F0 onset, midpoint, and offset values) of tone productions was noted. (2) Balkenhol et al. focused on the benefits of adding a hearing aid (HA) on the contralateral side of a CI for sentence recognition and auditory evoked potential (AEP) responses. Their results suggest that bimodal listeners can take advantage of head shadow and binaural summation effects, but cannot make use of binaural squelch and spatial release from masking at 6 months post-CI. The perceptual benefit of bimodal hearing may be objectively evaluated by the AEP responses, supported by a significant correlation of binaural summation effects with AEP latency differences between the bimodal and CI-only conditions. (3) Matz et al. studied auditory stream segregation in CI users by changing the spectral- and amplitude-modulation rate of narrowband noise (NBN) bursts. Their results showed the deficits of CI users in segregating NBN bursts into different auditory streams when they were moderately separated in the spectral domain compared to NH listeners. Both groups were able to utilize build-up effects to segregate auditory streams when lengthening the duration of stimulus sequences. (4) Liang et al. investigated the effect of implantation side on CI outcomes through behavioral measures and brain activation measured by ACC. Their results indicated that the implantation side may affect neural plasticity patterns in the adult population, demonstrated by a unique correlation between ACC activation patterns and performance in frequency change detection in subjects with a right-ear CI. (5) Yao et al. presented a literature review on the research status and future development of cochlear reimplantation. Although techniques are relatively matured since the 1980s, several issues were identified by the authors. The need for an international consensus statement on cochlear re-implantation in relevant problems (e.g., to standardize the definition, calculation formulas of reimplantation rate, and follow-up systems) is highlighted. (6) Wang et al. studied changes in vestibular function in patients who received a minimally invasive CI surgery 1 year before the study. The functions of semicircular canals and otolith were assessed by a comprehensive battery of tests. Most of the vestibular functions could be preserved with no damage discrepancy among the otolith and three semicircular canal functions at 12 months post CI. (7) Di Nardo et al. explored the benefits of CI in tinnitus suppression. Single-channel stimulation resulted in a significant reduction of tinnitus loudness. Their results provided insights into the mechanisms of tinnitus and the development of tinnitus therapies. (8) Bissmeyer et al. examined the effect of a computer-based musical training program on musical interval identification of CI users and listeners with no known hearing loss and the correlation between low-level psychophysics and higher-level musical abilities. They observed strong correlations between pitch sensitivity and musical interval identification for the two participant groups. However, the effect of the training program in this study on musical interval identification was small among CI users. The authors discussed directions toward improving pitch access and auditory training of musical interval appreciation for CI users.

The papers presented in this Research Topic provide a snapshot of the latest discoveries in the benefits and outcomes of CI. We believe that this Research Topic provides an interdisciplinary forum for researchers working in the fields of speech and hearing science, computational neuroscience, medicine, and biomedical engineering to present the most recent ideas, methodologies, and studies for understanding and improving the benefits and outcomes of CI. Future studies on the benefits and outcomes of CI could continue in several aspects, including establishing neural biomarkers of central auditory processing, discovering new predictive factors of CI outcomes, and exploring CI benefits in speech production, emotion processing and spatial listening. Utilizing multi-disciplinary methods in future CI studies is also recommended, e.g., combining neuroimaging and psychophysical tools to assess CI outcomes and integrating traditional statistical tests and artificial intelligence algorithms to analyze clinical findings.

The editors sincerely thank all the authors, reviewers, and Frontiers editors as well as the staff involved in the preparation of this Research Topic.

## Author contributions

FC, JC, and XL contributed to the drafting and revision. All authors contributed to the article and approved the submitted version.

## Conflict of interest

The authors declare that the research was conducted in the absence of any commercial or financial relationships that could be construed as a potential conflict of interest.

## Publisher's note

All claims expressed in this article are solely those of the authors and do not necessarily represent those of their affiliated organizations, or those of the publisher, the editors and the reviewers. Any product that may be evaluated in this article, or claim that may be made by its manufacturer, is not guaranteed or endorsed by the publisher.

